# Myxedema Coma Complicated by Pancytopenia

**DOI:** 10.1155/2019/2320751

**Published:** 2019-07-14

**Authors:** Charles T. Mupamombe, Felix M. Reyes, Derek B. Laskar, Joseph Gorga

**Affiliations:** ^1^SUNY Downstate Medical Center, Department of Medicine, Brooklyn, NY, USA; ^2^SUNY Downstate Medical Center, Department of Pathology, Brooklyn, NY, USA; ^3^Kings County Hospital Center, Department of Pulmonary and Critical Care, Brooklyn, NY, USA

## Abstract

Hypothyroidism is common, with an extreme manifestation of myxedema coma if untreated. Hematologic consequences of myxedema coma include mild leukopenia and anemia, rarely pancytopenia. We present a patient with typical symptoms of myxedema coma, but found to be pancytopenic, with sustained response to levothyroxine and blood transfusion for anemia.

## 1. Background

The American Thyroid Association estimates more than 12% of the US population will develop a thyroid condition and 20 million people have some form of thyroid disease. More than half of these people will be undiagnosed [1]. Hypothyroidism is the most common thyroid disorder. Symptoms usually include tiredness, mental depression, sluggishness, cold intolerance, weight gain, dry skin and hair, constipation, and menstrual irregularities in women [2]. The most extreme manifestation of untreated hypothyroidism is myxedema coma, which carries a mortality rate as high as 25–60% [3]. A few cases in the literature have been reported of hematologic manifestations due to hypothyroidism, which resolved following adequate thyroid hormone replacement [4–7]. We present a case of a patient whose initial known thyroid disorder presentation was myxedema coma, with a complication of pancytopenia.

## 2. Case

A 71-year-old female who had a reported history of dementia and depression, being treated with carbidopa-levodopa, memantine, quetiapine, selegiline, and sertraline, was brought in by her family for refusal to eat, walk, or talk. The family of the patient endorsed that the patient had gone from hospital to hospital and always been diagnosed with dementia. Her prescriptions were always obtained from emergency departments. A primary care physician was not provided, and it was not clear who had initially prescribed these medications.

Her vital signs were significant for hypothermia, with a temperature of 91°F (32.8°C); she had bradycardia at 33 beats per minute; oxygen saturation by pulse oximetry was 90% on room air; and her initial blood pressure was 128/72 mmHg. On physical examination, she was contracted and cachectic but withdrawing to pain, she had a regular rhythm and rate of the heart, and her extremities were nonedematous. There was evidence of hair loss on her head. The rest of her physical examination was unremarkable.

While in the emergency department, her systolic blood pressure decreased to 60 mmHg, unresponsive to aggressive resuscitation with intravenous fluids. The Emergency Room physicians treated her with vasopressors to maintain an adequate mean arterial pressure for perfusion. In addition, her mental state deteriorated further, becoming increasingly altered and eventually falling into a soporous state. She was intubated for airway protection. She was transferred to the Medical Intensive Care Unit for further care and evaluation. Her home medications were not continued at this point.

Her initial complete blood count (CBC) was significant for white blood cell count (WBC) of 1.4/nL (absolute neutrophil count (ANC) of 1086 × 10^3^/*μ*L), hemoglobin (Hb) of 12.3 g/dL, hematocrit of 33%, platelet count of 104/nL, and MCV of 79.5 fL (78–95). Her reticulocytes were 0.51%, with a reticulocyte index of 0.30 (using the lower limit normal hematocrit for our lab of 37%). During the first week of admission, her WBC reached a nadir of 0.76/nL (ANC 496 × 10^3^/*μ*L) and Hb nadir of 6.6 g/dL, without any evidence of hemorrhage identified. Her platelets reached a nadir of 48/nL. Her creatinine level was 0.34 mg/dL, with a blood urea nitrogen level of 25 mg/dL. Our theory at the time was hemoconcentration, with administration of fluid subsequently causing a dilution effect. This was suggested by her serum total protein decrease from 6.1 g/dL (6–8.5) on arrival to 4.3 g/dL after continuous fluid resuscitation. Her creatine kinase (CK) level was elevated, measuring 687 U/L (20–180).

Her thyroid-stimulating hormone was 24.5 miU/L (0.27–4.20); free thyroxine was measured at 0.886 ng/dL (0.93–1.70); total thyroxine (T4) was 3.7 *μ*g/dL (4.5–11.7); free triiodothyronine (T3) was 0.989 pg/mL; and total T3 was 36.31 ng/dL (80–200). Her PM random cortisol level was 16.2 *μ*g/dL (AM range 6.2–19.54; PM range 2.3–11.9). Her thyroid-stimulating immunoglobulin was 55% (0–139%), and T3 uptake was 37.45% (28–41%).

She had a serum sodium level of 114 mmol/L (136–146) and serum potassium level of 4.6 mmol/L (3.5–5.0). Her serum osmolality was 245 mOsm/L (275–295), urine sodium 68 mmol/L, and urine osmolality 401 mOsm/L (300–1000). Her AST level was elevated to 96 U/L (10–35) and ALT 72 U/L (0–31). Her urinalysis was not suggestive of an infectious process. Her venous lactate reached a high level of 2.7 mmol/L (0.5–2.2), but decreased to normal with hydration and remained as such throughout her stay in the MICU. Urine toxicology was negative. Blood culture and urine culture were unremarkable for any organisms. See Table 1 for a summary of selected results.

**Figure 1:**
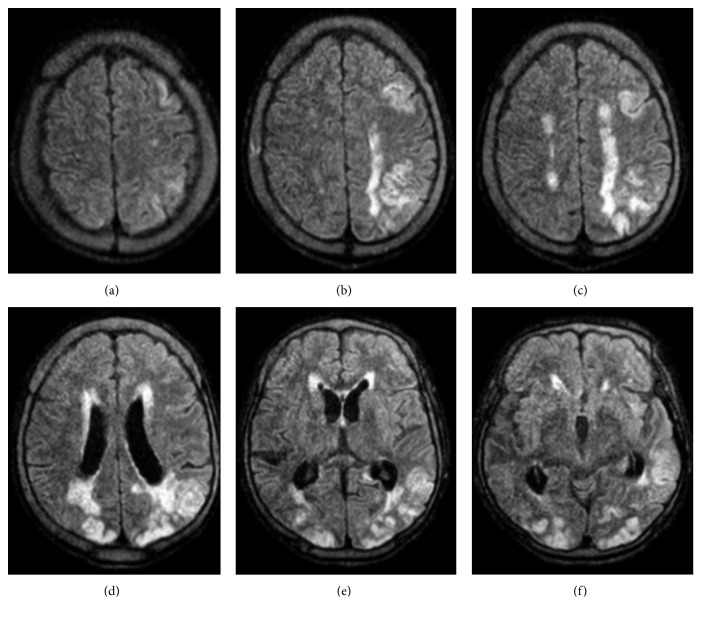
T2-weighted MRI without contrast images of the brain. The hyperintense areas reflect areas of ischemia. These were reviewed as recent infarcts in the ACA, MCA, and PCA territories. No intracranial hemorrhage can be identified. The radiologist also noted a mild mass effect with no midline shift.

**Figure 2:**
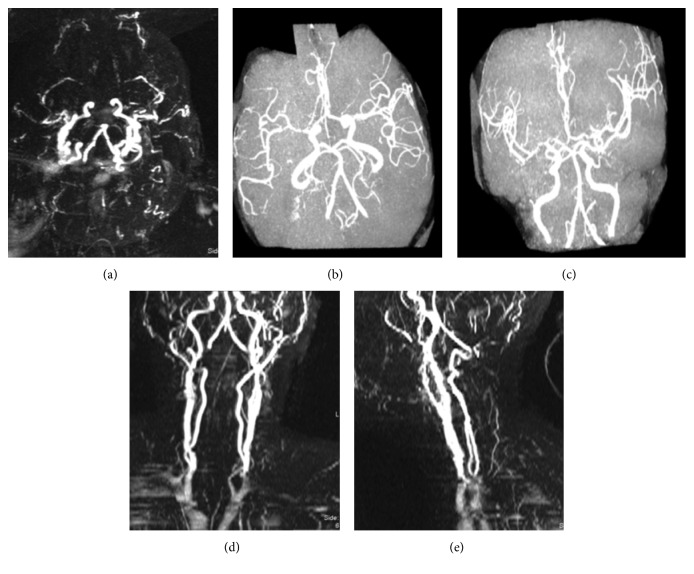
Magnetic resonance angiography (MRA) images of the head and neck are shown. The top 3 panels show MRA of the head, reviewed as multifocal areas of irregularity and narrowing within the anterior and posterior circulations, including pronounced stenosis of the bilateral carotid termini, middle cerebral arteries, right ACA A1 segment, and right PCA P3 and P4 segments with nonvisualization of the mid to distal right and P4 segment. The bottom panels show MRA of the neck, which was reviewed as unremarkable.

A chest X-ray obtained was unremarkable. Computed tomography (CT) of the head without contrast revealed a prominent bilateral, symmetric occipital-parietal white matter hypodensity with preserved overlying gray matter attenuation without significant local mass effect. The reviewing radiologist suggested this could be related to posterior reversible encephalopathy syndrome, sequela of hypoglycemia, or progressive multifocal leukoencephalopathy (PML) in the proper clinical setting. A magnetic resonance image (MRI) of the brain without contrast was obtained due to a continued nonresponsive state and to further investigate the results of the CT of the head. This revealed multifocal areas of recent ischemic infarction involving large areas of the bilateral posterior cerebral artery (PCA) territories, the left middle cerebral artery (MCA) territory, and small infarcts in the right anterior cerebral artery (ACA) and bilateral watershed areas (Figures 1 and 2).

**Figure 3:**
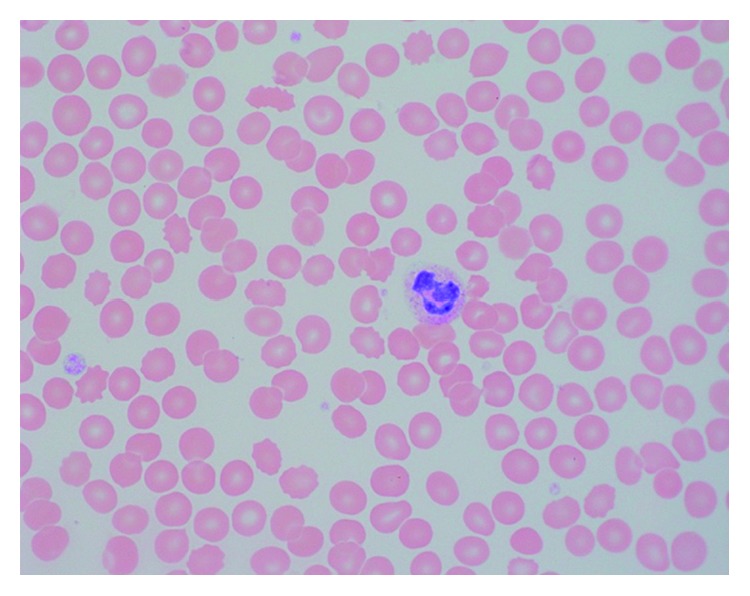
Peripheral blood smear shows pancytopenia with paucity of leukocytes, erythrocytes, and platelets. A neutrophil with cytoplasmic toxic granulations and adjacent platelet is seen (Wright–Giemsa, original magnification × 100).

The probability of myelodysplastic syndromes (MDSs) was high, so we sought a bone marrow biopsy for further investigation. However, her family opted not to have this procedure performed. We decided to obtain a peripheral blood smear and flow cytometry; in the meantime, this gave the family more time to decide on goals of care. The peripheral blood smear revealed decreased white blood cells, few neutrophils which were hypolobulated and hypergranular; normocytic normochromic red blood cells, rare polychromasia without tear drop cells; and decreased platelets, without clumping (Figure 3). Serum electrophoresis for gamma globulins was within normal ranges. Flow cytometry returned as normal. Serum vitamin B12 and folate were within acceptable range.

She was treated with normal saline and empiric broad-spectrum antibiotics. Thyroid myxedema was the most likely differential, so she was started on hydrocortisone and intravenous levothyroxine. Her WBC count improved to 5.43/nL 9 days after initiation of levothyroxine. Her platelets also improved to a count within a normal laboratory range of 148/nL 13 days after initiation of levothyroxine. For unclear reasons, she had a sudden decline in hemoglobin on day 18 to a level of 5.7 g/dL, which was confirmed on repeat CBC. She received 2 units of packed red blood cells (PRBCs) as treatment. There was no evidence of hemolysis on her lab values or signs of any hemorrhage. Her Hb improved to 10.2 g/dL after transfusion and remained between 9 and 10 g/dL for the remainder of her admission, without further need for transfusion throughout her stay. Unfortunately, our patient did not return to a conscious state and expired after 32 days as an inpatient.

## 3. Discussion

Our differential diagnosis for the cause of the hematologic disturbances in our patient included hypothyroidism, sepsis, drug-induced hemorrhage, and nutritional deficiencies. We will discuss in brief some of them below based on a brief literature review.

The pathogenesis of anemia in hypothyroidism may be related to decreased oxygen requirement due to a decrease in the basal metabolic rate [6]. Pancytopenia due to marrow hypoplasia has been hypothesized and reported in patients with myxedema coma but does not seem to be commonplace [6, 8]. If it occurs in uncomplicated hypothyroidism, anemia is characteristically normochromic and normocytic, less common is macrocytic or microcytic [6]. Some sources report the incidence of anemia in uncomplicated hypothyroidism to be slightly less than 30% [6]. Patients with hypothyroidism are known to have reduced levels of erythropoietin, with one theory being due to reduced renal perfusion [9].  Table 2 summarizes some cases found in the literature linking pancytopenia to hypothyroidism. Interestingly, all cases found were of female patients.

Hemorrhagic shock has been shown to suppress early and late progenitor cell growth in rats [12]. We are unsure if the same link exists in humans. Splanchnic hypoperfusion may play a role in bone marrow ischemia as proposed by some investigators [13]. Other signs of systemic hypoperfusion would be expected to be seen, such as ischemic hepatitis, due to profound hypotension [14]. The serum lactate level would also be predicted to be significantly elevated in such patients [15].

Medications should always be on the differential for potential causes of bone marrow injury. Memantine is 45% protein bound, undergoes partial hepatic metabolism, and has 74% renal excretion of its 3 minimally active metabolites, with an elimination half-life of 60–80 h [16–18]. Quetiapine is 83% protein bound, primary hepatically metabolized, and has 73% renal excretion, with a half-life of approximately 6-7 h [16–18]. Both memantine and quetiapine are metabolized via the cytochrome P450 family of enzymes. Memantine may be associated with agranulocytosis, leukopenia, pancytopenia, and thrombocytopenia according to postmarketing surveillance in a report by the United States Food and Drug Administration (FDA) [17, 19]. Quetiapine is also associated with neutropenia in postmarketing surveillance data [19]. According to the FDA, data disclosing the effects of both quetiapine and memantine on white blood cells are lacking, and the frequency of these effects could not be determined.

Medication-induced pancytopenia could have played a role for our patient; however, the relatively short period of time that her cell counts improved upon discontinuation of possible offenders made this unlikely. The long half-life of memantine could mean continued pharmacologic effect even with acute cessation; however, we would theorize a longer time for cell counts to improve than what we observed. However, the same argument cannot be applied for quetiapine due to a shorter half-life. In addition, the lack of a defined frequency via postmarketing data suggests that these side effects for both medications are extremely rare, and more studies are needed to confirm the link. We would also expect a cocktail of the more common side effects to have been observed at presentation, reversing upon cessation of medications, but this was not the case.

Cytopenias due to nutritional deficiencies, including folate and vitamin B12, have been well described in the literature, with marked improvement upon administration [11, 20–22]. In the case of folate and vitamin B12, there is an arrest in development in all cell lines, leading to decreased cell counts [20]. This is of particular concern in the elderly who are at increased risk of malnutrition. McHamon and Kamath reported a case of pancytopenia associated with vitamin B12 deficiency secondary to pernicious anemia, which improved upon vitamin B12 supplementation [11]. This etiology is reversible upon administration of the deficient nutrient; therefore, it is important for clinicians to check the levels of folate and vitamin B12 whenever a deficiency is suspected.

Our patient presented with multiple signs associated with myxedema coma: alopecia, bradycardia, altered mental state, hypotension, hyponatremia, elevated CK, initial mild leukopenia, and initial mild anemia [23]. In hindsight, the precipitating event could have been the stroke found on subsequent MRI of the brain, as this has been reported to induce myxedema coma [23]. She may have been among the 20 million estimated not to be aware of their thyroid condition, which was brought to light on this admission in its extreme manifestation. With our patient in the geriatric range, her hypothyroid probably presented in an atypical fashion [23], with decreased mobility and poor cognition, inappropriately diagnosed as dementia.

Our patient presented with hypotension, requiring vasopressor support to maintain perfusion. However, she did not exhibit a markedly impressive sign of hypoperfusion via serum lactate, which made bone marrow failure secondary to shock (hypoperfusion) lower on our differential. This lack of lactate production may be explained by decreased tissue metabolic demand secondary to decreased thyroid hormone. Her reticulocyte index was low, suggesting inadequate bone marrow response to anemia. Here, we would hypothesize this to be secondary to a lack of thyroid hormone.

Hypothermia, decreased WBCs, and hypotension can be seen in septic patients. In addition, the hypergranulations found in the neutrophils could be seen in an infection in some cases, but can be nonspecific. What argued against an infective process in our patient are the negative blood and urine cultures.

She had a significant decrease in the hemoglobin level, requiring transfusion of red blood cells. This fact is a confounder in whether her red blood cells would have improved on thyroid supplementation alone or not; however, it was deemed necessary to transfuse her at the time to try to improve her clinical status. What favors the argument that thyroid hormone played a significant role is that she did not require further transfusions throughout her admission as blood transfusion was administered relatively early in her clinical course. We suspect it may have been too early to have seen the effects of the thyroid hormone replacement. Her response to the blood transfusion was also very robust and unexpected, increasing from 6.6 g/dL to 10.2 g/dL after only just 2 units of packed red blood cells. It is not clear why this was observed; perhaps, the transfusion improved perfusion to her kidneys and, in turn, improved her erythropoietin production, sustaining her hemoglobin level from then on. She remained anemic but stable, which is an expected parameter in hypothyroidism.

A bone marrow biopsy would have helped in the diagnosis of her hematologic pathology; however, it was not ethically justified; the family wishes had to be respected. Moreover, a normal flow cytometry with normalizing white cell count on repeat testing left little support for its pursuit, especially given her continued soporous state. Although mild leukopenia and mild anemia can be present in patients with myxedema [23], having them occur simultaneously to the levels seen in our patient, along with added thrombocytopenia, makes our case unusual. This could support the theory of bone marrow hypoplasia in severe hypothyroidism.

With all the data that were collected and available to us, we believe the patient had pancytopenia secondary to hypothyroidism which presented as myxedema. This is supported by her cell counts normalized soon after administration of intravenous levothyroxine. Our case highlights the importance of maintaining hypothyroidism on the differential for reversible causes of anemia and dementia from a primary care perspective, as it appears she had moved from provider to provider without this being addressed. From a critical care point, pancytopenia should be managed as a part of a multisystemic complication of myxedema coma, knowledge of which can help guide management for critical care providers.

## Figures and Tables

**Table 1:** Summary of selected results.

Day 1 (range)	Day 3	Day 9	Day 13	Day 20
WBC 1.4/nL (4.5–10.9)	WBC 0.76/nL	WBC 5.43/nL	WBC 5.64/nL	WBC 8.49/nL
Hb 12.3 g/dL (12–16)	Hb 9.2 g/dL	Hb 8.1 g/dL	Hb 7.1 g/dL	Hb 9.1 g/dL
Hct 33% (37–47)	Hct 27.1%	Hct 22.6%	Hct 21.8%	Hct 27.2%
Plt 104/nL (130–400)	Plt 46/nL	Plt 85/nL	Plt 148/nL	Plt 388/nL
Na 114 mmol/L (136–146)		Na 141 mmol/L		
K 4.6 mmol/L (3.5–5.0)		K 3.4 mmol/L		
Cl 78 mmol/L (98–106)		Cl 100 mmol/L		
BUN 25 mg/dL (8–23)		BUN 22 mg/dL		
Cr 0.34 mg/dL (0.50–0.90)		Cr 0.43 mg/dL		
TSH 24.5 miU/L (0.27–4.20)			TSH 4.82 miU/L	
Free T4 0.886 ng/dL (0.93–1.70)			Free T4 0.478 ng/dL	
Total T4 3.7 *μ*g/dL (4.5–11.7)			Total T4 2.34	
Free T3 0.989 pg/mL				
Total T3 36.31 ng/dL (80–200)				

WBC, white blood cells; Hb, hemoglobin; Hct, hematocrit; Plt, platelet; Na, sodium; K, potassium; Cl, chloride; BUN, blood urea nitrogen; Cr, creatinine; TSH, thyroid-stimulating hormone; T4, thyroxine; T3, triiodothyronine.

**Table 2:** Some reported cases of pancytopenia associated with hypothyroidism found in the literature [4–6, 8, 10, 11].

Author(s)	Age	Sex	Presentation	Cell count	Treatment	Outcome
McMahon and Kamath [11]	25	F	Known hypothyroidism, iron deficiency anemia, fatigue, weight loss	WBC 3800/*µ*L	Levothyroxine + vitamin B12	Complete resolution of pancytopenia on 2-month follow-up
				Hemoglobin 6.9 g/dL		
				Platelet 158 × 10^9^/L		
Rathi and Peacey [6]	64	F	No past medical history, 4-week history of bilateral leg swelling, tiredness, dry skin	WBC 2900/*µ*L	Levothyroxine	Complete resolution of proteinuria and pancytopenia on 3-month follow-up after discharge
				Hemoglobin 10.2 g/dL		
				Platelet 127 × 10^9^/L (150–400)		
Tsoukas [4]	82	F	Confusion, lethargy, bradycardia, hypothermia, respiratory stridor, no past medical history	WBC 3990/*µ*L	IV hydrocortisone + IV levothyroxine, subsequent PO levothyroxine on discharge	Complete resolution of pancytopenia on 4-week follow-up after discharge
				Hemoglobin 8.5 g/dL,		
				Platelet 27 × 10^9^/L		
Song et al. [8]	68	F	Increasing immobility over 12 months, alopecia, drowsy, slurred speech, deep voice, no other past medical history	WBC 1600/*µ*L	IV hydrocortisone + IV triiodothyronine, oral levothyroxine replacement 1 week later	Resolution of white cell count and platelet count at 6 weeks on discharge Improved hemoglobin at 4-month follow-up after discharge
				Hemoglobin 8.2 g/dL		
				Platelet 35 × 10^9^/L		
Lee AC [5], letter to the editor	11	F	Patient developed postablative hypothyroidism and pancytopenia after radiotherapy for suprasellar germinoma	Absolute neutrophil 0	Packed red blood cell and platelet transfusions, thyroxine replacement + hormonal replacement	Pancytopenia resolved within a week of thyroid hormone replacement
				Hemoglobin 6.9 g/dL		
				Platelet 8 × 10^9^/L		
Shaaban et al. [10]	57	F	Restlessness, increased fatigue, weight gain, puffy face	WBC 2500/*µ*L	Initially IV and PO levothyroxine, discharged on PO only	White cell count improved after 1 week of thyroid replacement, at 3-month follow-up, her hematologic counts were stable
				Hemoglobin 8.4 g/dL		
				Platelet 95 × 10^9^/L		

## Additional Points

Hypothyroidism is common and should be considered as part of the differential diagnosis in patients with abnormal hematologic parameters. Pancytopenia can be part of a multisystemic complication of severe hypothyroidism.
